# Introduction of the Capsules environment to support further growth of the SBGrid structural biology software collection

**DOI:** 10.1107/S2059798324004881

**Published:** 2024-06-04

**Authors:** Carol Herre, Alex Ho, Ben Eisenbraun, James Vincent, Thomas Nicholson, Giorgos Boutsioukis, Peter A. Meyer, Michelle Ottaviano, Kurt L. Krause, Jason Key, Piotr Sliz

**Affiliations:** a Department of Biological Chemistry and Molecular Pharmacology, Harvard Medical School, Boston, Massachusetts, USA; b Department of Biochemistry, University of Otago, Dunedin, New Zealand; c Department of Pediatrics, Boston Children’s Hospital, Boston, Massachusetts, USA; University of Oxford, United Kingdom

**Keywords:** structural biology, scientific software, FAIR principles, high-performance computing, structure determination, SBGrid, Capsule Software Execution Environment

## Abstract

The expansive scientific software ecosystem, characterized by millions of titles across various platforms and formats, poses significant challenges in maintaining reproducibility and provenance in scientific research. The diversity of independently developed applications, evolving versions and heterogeneous components highlights the need for rigorous methodologies to navigate these complexities. In response to these challenges, the SBGrid team builds, installs and configures over 530 specialized software applications for use in the on-premises and cloud-based computing environments of SBGrid Consortium members. To address the intricacies of supporting this diverse application collection, the team has developed the Capsule Software Execution Environment, generally referred to as Capsules. Capsules rely on a collection of programmatically generated bash scripts that work together to isolate the runtime environment of one application from all other applications, thereby providing a transparent cross-platform solution without requiring specialized tools or elevated account privileges for researchers. Capsules facilitate modular, secure software distribution while maintaining a centralized, conflict-free environment. The SBGrid platform, which combines Capsules with the SBGrid collection of structural biology applications, aligns with FAIR goals by enhancing the findability, accessibility, interoperability and reusability of scientific software, ensuring seamless functionality across diverse computing environments. Its adaptability enables application beyond structural biology into other scientific fields.

## Abstract 2

The integration of Capsules within the SBGrid software-management platform marks a pivotal advancement in addressing the challenges of scientific software distribution, dependency management and computational reproducibility.

## 1. Introduction

Researchers in various scientific fields have access to a software ecosystem with millions of titles accessible through websites and repositories (Dey *et al.*, 2021 ▸; Pietri *et al.*, 2019 ▸). An example is the Elixir ‘bio.tools’ registry (Ison *et al.*, 2019 ▸), which lists over 43 000 software applications for life sciences in 2023. As a result, biologists are frequently confronted with the challenge of navigating an array of applications and complex systems when selecting software tools that are suitable for a particular project. Access to adequate software metadata as well as the ability to rapidly install the latest version of the most suitable application are critical and can minimize non­scientific factors that influence scientists’ selection of software.

Software applications for biomedical research are developed globally primarily by scientists (Morin & Sliz, 2013 ▸) who are embedded at research institutions. Typically these applications evolve through many versions over time, and have diverse underlying components. They are implemented in various programming languages, libraries and frameworks, each with its own set of dependencies (Mayer & Bauer, 2015 ▸; Tomassetti & Torchiano, 2014 ▸). The complex web of dependency management that results must be approached carefully to guarantee compatibility and reproducibility (Baresi *et al.*, 2024 ▸; Fan *et al.*, 2020 ▸; Babinet & Ramanathan, 2008 ▸; Belguidoum & Dagnat, 2007 ▸), and to prevent excessive support overhead, which can consume up to 30% of the time spent in computational projects (Kumfert & Epperly, 2002 ▸; Hochstein & Jiao, 2011 ▸). The variability of computational resources deployed in support of biomedical research adds a significant layer of complexity; software applications are rarely distributed in a format that can be immediately executed on researchers’ computers. Before each software application is used it typically needs to be compiled and configured, as well as tested on a specific operating system (OS) and hardware configuration. In particular, many recently developed applications that rely on artificial intelligence (AI) approaches (Pandey *et al.*, 2022 ▸) must also be optimized to support parallel computing and graphics processing unit (GPU) acceleration. The challenges faced in navigating the complex landscape of scientific software highlight the need for systematic approaches to versioning, compatibility and performance optimization (Morin & Sliz, 2013 ▸).

Navigating the landscape of scientific software is further complicated by a lack of uniformity in software access (Morin *et al.*, 2012 ▸). Software applications can be distributed through various channels such as package managers, port systems, container registries, laboratory or project websites, and development version-control repositories, each with its own set of requirements and limitations. For example, containers provide isolated environments for applications while sharing the host OS kernel (Bakshi, 2017 ▸; Casalicchio & Iannucci, 2020 ▸), but can be complex to orchestrate and require specialized knowledge to secure, manage and run (Bui, 2015 ▸; Combe *et al.*, 2016 ▸). Project websites and version-control repositories often provide access to the most recent versions of applications, whereas software is often distributed as a source code, which necessitates a complicated, OS-specific compilation process that requires technical expertise beyond the capability of many scientists. System packages and port systems handle dependencies and versioning, but software installation is managed at the OS level (Gamblin *et al.*, 2015 ▸) and often there is a delay before the most recent version of an application is included.

The complexity of software maintenance and distribution can make it even more problematic to address the needs for computational reproducibility and provenance in scientific research. Inconsistencies in computational platforms, such as software, databases, compilers, external libraries, hardware and OSs, can lead to different results even when identical versions of software are used under different settings (Tommaso *et al.*, 2017 ▸; Harris *et al.*, 2008 ▸; Ivie & Thain, 2018 ▸; Heil *et al.*, 2021 ▸; Vitek & Kalibera, 2011 ▸; Morin *et al.*, 2012 ▸). Addressing these issues is a necessity for transparent and validated models, particularly in critical fields such as healthcare, where outcomes are paramount (Eddy *et al.*, 2012 ▸). A survey in the journal *Nature*, with 1576 researchers, revealed that reproducibility continues to be an ongoing challenge for scientists. Notably, 52% of participants admitted that they could not reproduce their own experiments, and more than 70% failed to replicate the work of others (Baker, 2016 ▸). While technology has enabled large data collection and sophisticated algorithms, it also poses challenges for the independent verification of findings (National Academies of Sciences, Engineering and Medicine, 2019 ▸; Peng & Hicks, 2021 ▸). There is still a need for standardized and reproducible ways to deploy software to further improve research reproducibility (Mesirov, 2010 ▸; Joppa *et al.*, 2013 ▸).

Structural biology is a field of research that is conducted through the intersection of several scientific disciplines: biology, physics, mathematics and computer science. It relies on many specialized software tools, each designed to solve specific aspects of structure-determination problems (Morin & Sliz, 2013 ▸). The SBGrid initiative (or SBGrid) develops a platform that simplifies software use in structural biology (Morin *et al.*, 2013 ▸). It started in 2001 to support X-ray crystallography at Harvard University (Jin *et al.*, 2003 ▸; Sliz *et al.*, 2001 ▸, 2003 ▸) and by 2005 had expanded to support cryogenic electron microscopy (cryoEM) research at Harvard Medical School (Gonen *et al.*, 2004 ▸; Fotin *et al.*, 2004 ▸). The SBGrid platform was developed to install and upgrade a growing collection of structural biology software on on-premises and cloud-based computing resources supporting hundreds of structural biology laboratories: all members of the SBGrid Consortium. Under the original SBGrid Monolithic Software Execution Environment (MSEE), all applications in the SBGrid collection could be immediately executed without further configuration (Morin *et al.*, 2013 ▸). Additionally, the SBGrid team has established services to help researchers cite software (Socias *et al.*, 2015 ▸), execute large computational workflows on opportunistic resources of the Open Science Grid (O’Donovan *et al.*, 2012 ▸; Stokes-Rees & Sliz, 2010 ▸), access and archive data from synchrotron facilities (Stokes-Rees *et al.*, 2012 ▸) and publish experimental data sets through the SBGrid Data Bank (Meyer *et al.*, 2016 ▸). While developing these additional support modalities, the SBGrid team continued to address the challenges of scientific software management and use.

This manuscript details our recent efforts to further enhance the scalability and usability of the SBGrid platform. To address the evolving complexity and volume of structural biology software, we have developed and implemented a more scalable approach to scientific software management: the Capsules Software Execution Environment (CSEE) system, also known as Capsules. Capsules isolate each software application along with its dependencies, environment settings and configurations into a singular package. This design is underpinned by the Environment Definition (ED) files, which specify software dependencies, the Capsules Executable Mapper (CEM), which resolves namespace conflicts, and the Capsules Runtime (CR) scripts, which instantiate the environment and run the executable. Capsules are packaged with the SBGrid collection, thereby providing users with an out-of-the-box solution that requires no system-administration access and operates consistently across all hardware platforms. The implementation of Capsules facilitated the development of a new, modular SBGrid installation manager as well as the extension of SBGrid to support computational biology more broadly through the new BioGrids collection. Additionally and importantly, Capsules provide a foundation for additional improvements in the areas of research reproducibility and security, and align with FAIR software principles (Wilkinson *et al.*, 2016 ▸) at the installation and execution level.

## 2. Methods and results

### 2.1. SBGrid collection of scientific applications

**Figure 1:**
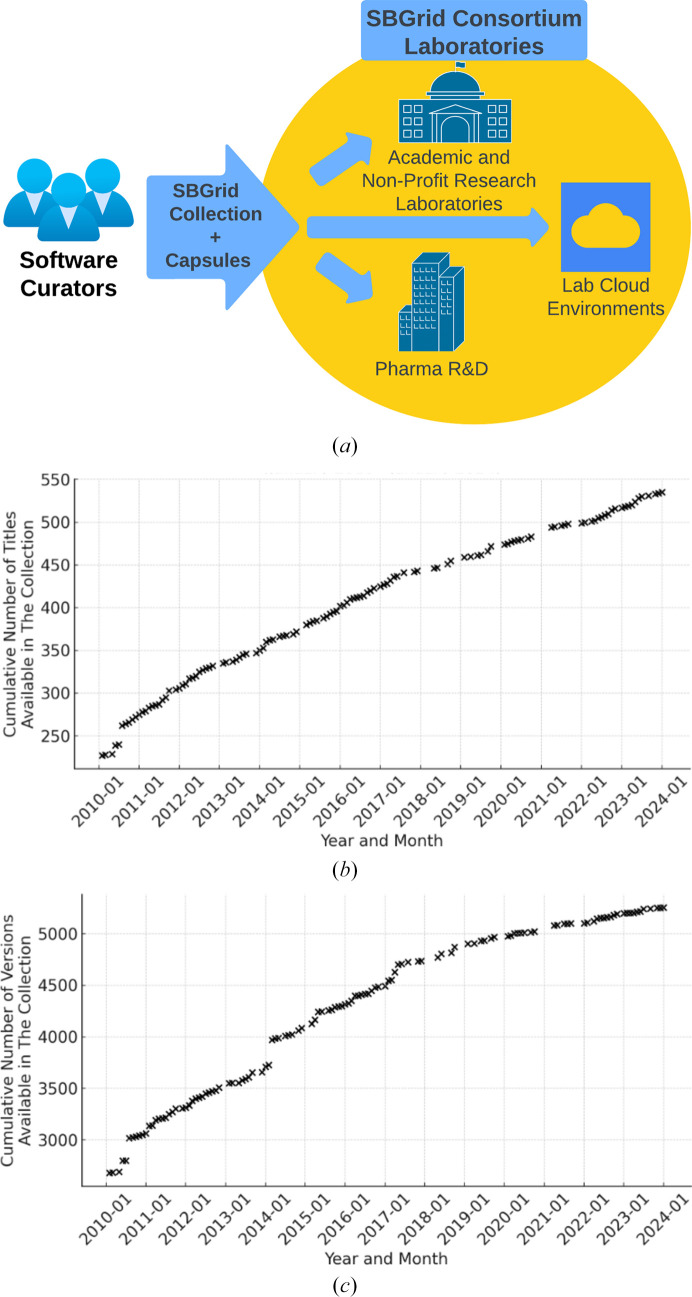
SBGrid platform and growth of the SBGrid collection. (*a*) The SBGrid platform was developed to install and upgrade a growing collection of structural biology software on computers in hundreds of academic and industrial structural biology laboratories, as well as their corresponding cloud computing resources. (*b*) Cumulative growth of software titles and (*c*) software versions in the 64-bit Linux and Intel/ARM macOS branches of the SBGrid collection. The 64-bit Linux and Intel/ARM macOS branches were established in 2003 and 2006, respectively, and gradually replaced the earlier 32-bit Linux, PowerPC-based Mac OS X and original SGI IRIX branches. Data for January 2024 were collected in February 2024, showcasing the most recent expansion of the collection.

Since our previous report (Morin *et al.*, 2013 ▸), we have developed SBGrid into a comprehensive platform focused on the nuanced demands of structural biology scientists (Fig. 1 ▸ *a*). The SBGrid collection has expanded to over 530 encapsulated, ready-to-run software titles, nearly doubling the size of the SBGrid collection (Fig. 1 ▸ *b*). These titles are distributed among Crystallography (87), NMR (48), CryoEM (144), Computational Chemistry (76), Structure Visualization and Analysis (105), and additional supporting applications (95). The SBGrid collection comprises two active branches, supporting 64-bit Linux and Intel/ARM macOS, as well as three older branches that are still preserved but are no longer maintained (32-bit Linux, PowerPC-based Mac OS X and the original SGI IRIX OS). Moreover, the 64-bit branch is used by Windows users through the Windows Subsystem for Linux (WSL) environment.

With the help of the SBGrid installation manager, the SBGrid collection is seamlessly installed and upgraded on on-premises and cloud-based computers supporting the SBGrid Consortium laboratories, where end users have zero-configuration access to multiple versions of software titles (Fig. 1 ▸ *c*). The growth trend of the SBGrid collection reflects the ongoing development of and updates to structural biology software applications, emphasizing the dynamic nature of structure-determination methods in the continually evolving and adapting field of structural biology.

Applications in the SBGrid collection can come with a plethora of individual executables with associated libraries, documentation and data. The total number of executables in the SBGrid collection exceeds 56 000, requiring approximately 2.3 TB of storage for Linux and 0.8 TB for macOS. The SBGrid collection of titles is linked to over 510 000 dynamic libraries tailored to the requirements of the individual application. Titles in the SBGrid collection are governed by their developer’s licensing terms, and the SBGrid collection includes over 220 titles under open-source licenses. These licenses include the Lesser General Public License (LGPL), the GNU General Public License versions 2 and 3 (GPLv2 and GPLv3, respectively), the Massachusetts Institute of Technology (MIT) License, the Creative Commons Restricted License and the Academic Free License (Supplementary Table S1). The remaining titles are licensed under a variety of academic licenses, and a few commercial titles are included as well (Morin *et al.*, 2012 ▸; Morin & Sliz, 2013 ▸). This extensive assortment reflects a commitment to accommodating various licensing requirements. Our unified licensing agreement simplifies licensing processes, which reduces the potential legal complexity for research-use purposes. SBGrid’s licensing strategy and its collaboration with developers from diverse backgrounds underscore our commitment to expanding impact, simplifying software distribution and ensuring licensing compliance for the software used in scientific research.

**Figure 2:**
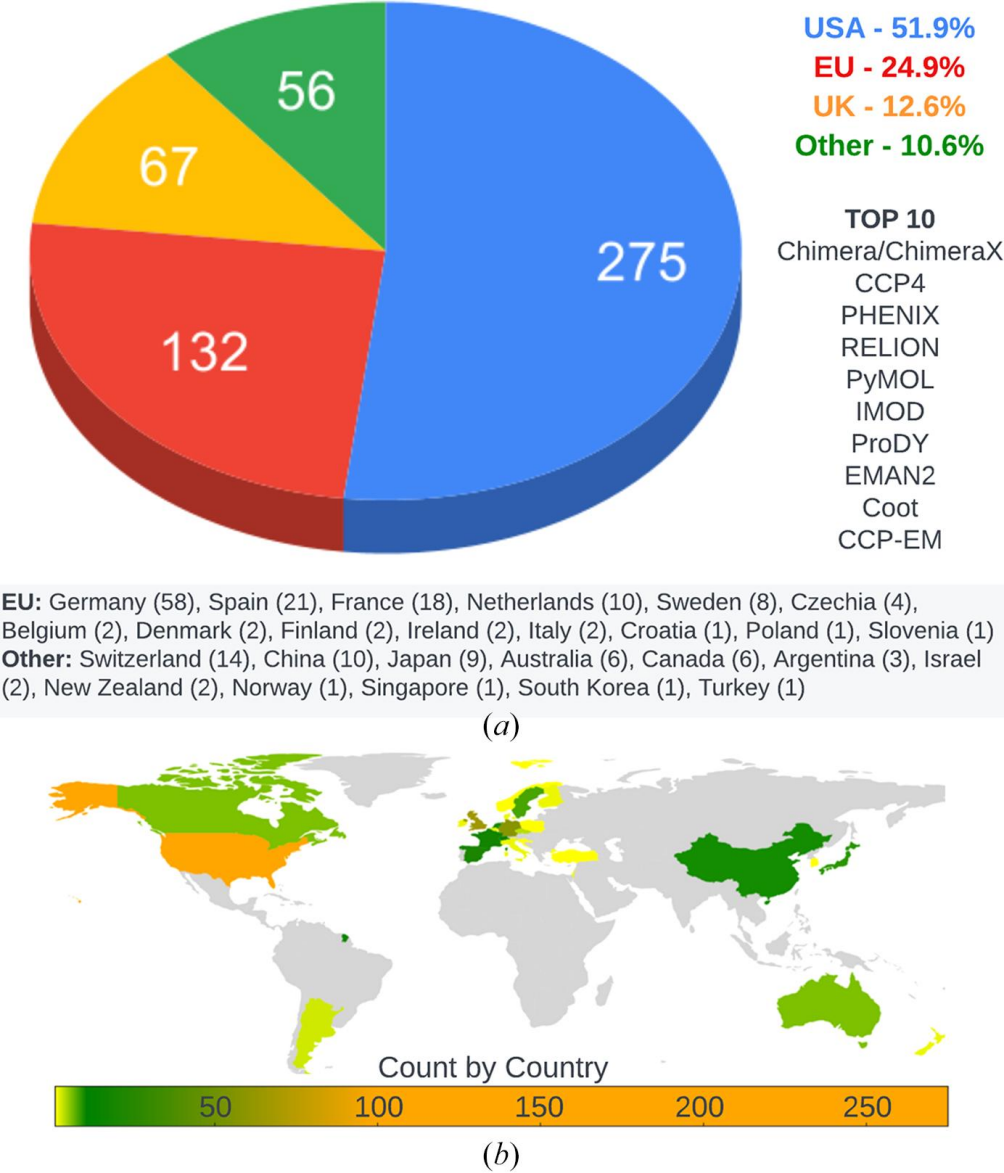
(*a*) Distribution of software titles within the SBGrid collection by country of origin and their percentage share within the SBGrid collection. The total number of titles developed within each country is indicated in parentheses. The top ten applications used from March 2023 to March 2024 were developed in the United Kingdom and the United States. The applications are ranked as follows: *UCSF Chimera*/*ChimeraX* (Pettersen *et al.*, 2004 ▸; Goddard *et al.*, 2018 ▸; US), *CCP4* (Agirre *et al.*, 2023 ▸; UK), *Phenix* (Liebschner *et al.*, 2019 ▸; US), *RELION* (Scheres, 2012 ▸; UK), *PyMOL* (Schrödinger; US), *IMOD* (Kremer *et al.*, 1996 ▸; US), *ProDY* (Bakan *et al.*, 2011 ▸; US), *EMAN2* (Tang *et al.*, 2007 ▸; US), *Coot* (Emsley *et al.*, 2010 ▸; UK) and *CCP-EM* (Wood *et al.*, 2015 ▸; Burnley *et al.*, 2017 ▸; UK). (*b*) A choropleth map illustrating the distribution of software title origins within the SBGrid collection, with color intensities reflecting the quantity of software originating or predominantly originating from each country. The map underscores the wide-ranging geographical origins of the software titles compiled in the SBGrid collection.

Structural biology software supported through the SBGrid collection has predominantly been developed in the US (52%), with significant contributions from the EU (25%), the UK (13%) and other regions (Fig. 2 ▸, Supplementary Table S1). Notably, the large and widely utilized application *CCP*4 (Agirre *et al.*, 2023 ▸), which has greatly influenced structural biology software-development efforts, was developed in the UK. The titles in the SBGrid collection were written in a variety of programming languages, such as C/C++, Fortran, Java, Python, Perl, R, Matlab and Tcl/Tk. The ten most frequented titles by unique users in 2023 utilize C++ (four titles), Python (four titles), C (one title) and Fortran (one title) for their core functionality (Supplementary Table S1).

The SBGrid team monitors for new versions, receives user requests and produces a monthly update of the SBGrid collection. In 2023, SBGrid released 16 titles per month on average, which include 12 new versions of existing applications and four new software titles. Applications are sourced from a diverse range of channels, including research websites, cloud storage solutions such as Google Drive, various package managers and version-control repositories such as GitHub, SourceForge and Bitbucket. The applications come in various formats, including tarballs, zip, jar, bin, install scripts and .git links pointing to specific Git repositories. In a systematic review of 50 titles randomly selected from the 345 used in the first eight months of 2023, we observed a distribution of download sources: approximately 50% of these titles provided links to research websites, 44% to GitHub repositories, 4% to package managers and 2% to Google Drive. It is important to note that many titles provide multiple download options; some titles use different download options for installable artifacts and the large data libraries required to use the software. Therefore, these percentages reflect the prominence of each source in the download instructions rather than in the strict exclusivity to a single download method. Additionally, the packaging formats of these 50 titles consisted of 44% in tarballs, 38% as Git repositories, 8% in package manager-specific formats, 6% in zip format and 2% each as jar and executable image formats (Supplementary Table S1). The diversity of formats and structural biology software distribution channels that SBGrid manages highlights the technical challenge that structural biologists without access to SBGrid must overcome.

### 2.2. Capsules

**Figure 3:**
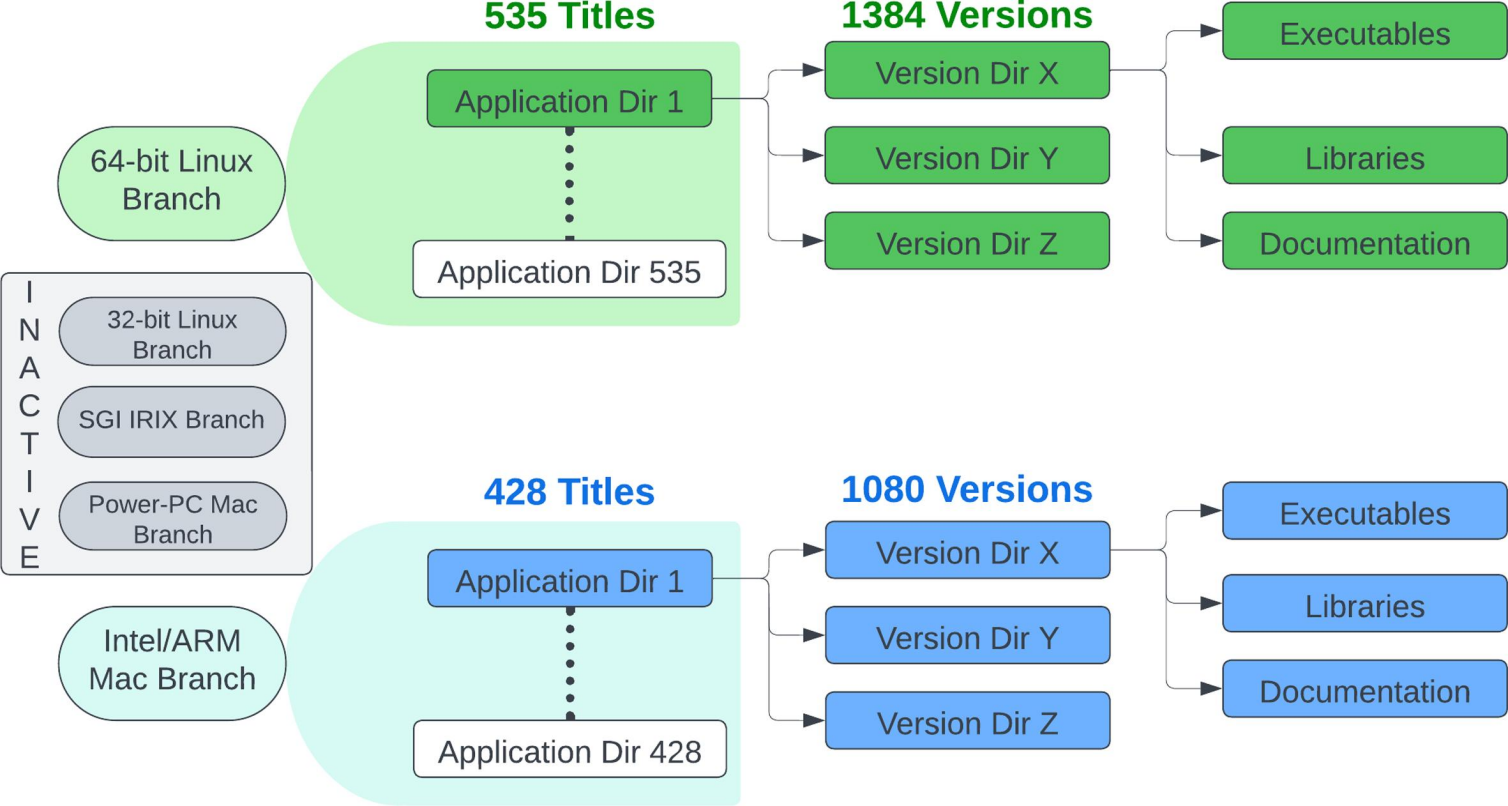
Two actively developed branches of the SBGrid collection support 64-bit Linux and Intel/ARM macOS OS. Within each branch, software is organized through application- and version-specific subdirectories.

To support streamlined, zero-configuration access to all software in the SBGrid collection, we developed the Capsules Software Execution Environment (CSEE), also referred to as Capsules. In the SBGrid collection, software is organized within OS- and architecture-specific software branches (Fig. 3 ▸). Two branches are continuously developed, one for 64-bit Linux and one for Intel/ARM Mac, and three older branches are still available for legacy OS. Within each SBGrid branch, each application is contained within a software title-specific directory, which is referred to as an Application Directory. Each Application Directory typically contains several Version Directories, which contain the executables, libraries, documentation and data files required to run the specific version. Many structural biology software titles are complex and additionally might depend on other specific versions of customized software titles. Supporting this convoluted network of dependencies eventually impaired our earlier Monolithic Software Execution Environment (MSEE) and led to the development of Capsules.

**Figure 4:**
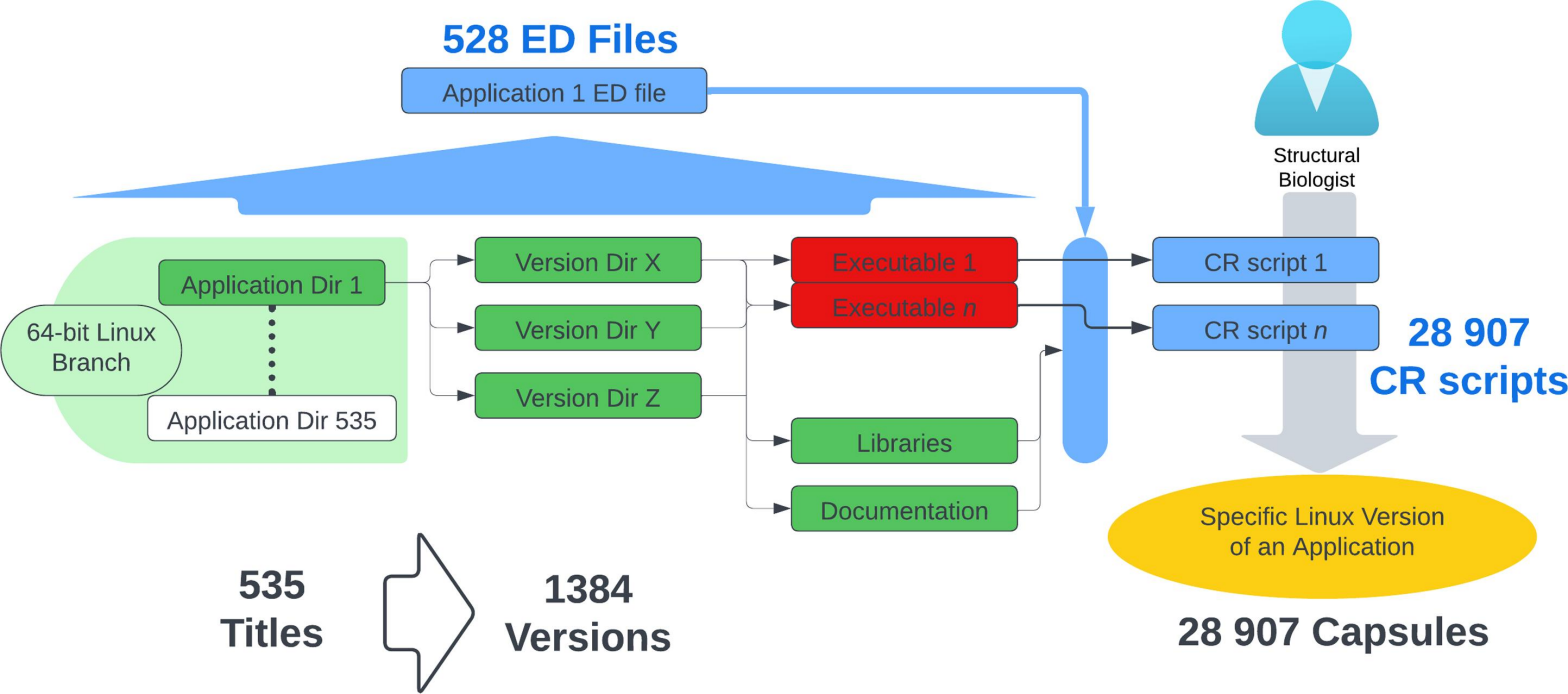
Modular packaging of the SBGrid collection. Each of the approximately 500 software titles is independently packaged into Capsules, isolating them from the broader system to prevent increasing complexity. The runtime environments are programmatically generated using ED files, ensuring a streamlined setup tailored to the specific needs of each software title, with exceptions noted for non-executable content.

With Capsules (Fig. 4 ▸) all software titles are packaged independently, isolating each from the broader system, thereby making the SBGrid collection modular and immune to increasing complexities. In this environment, structural biology executables are automatically run within a carefully optimized runtime environment, which includes libraries, environment variables and access to executables that support the particular application.

With approximately 500 software titles in each active software branch, SBGrid supports close to 50 000 programmatically generated CR scripts that are unique and specific to all executables in the SBGrid collection (typically with many executables per software title). These CR scripts (Fig. 4 ▸ and Supplementary Fig. S1*a*) provide users with immediate access to all required, version-specific libraries and other software dependencies.

The environment supporting CR scripts is generated through corresponding ED files (Fig. 4 ▸ and Supplementary Fig. S1*a*), streamlining the setup according to the requirements of each title. The only exceptions are a few titles that contain database files or code that is otherwise not executable. The ED files are manually written, edited and tested by the SBGrid software curators to capture the distinct dependencies and environment requirements of the contained executables. The curators use information from the application developer to determine the environment requirements of the application. The ED files often contain constructs that initiate the environment based on conditionals such as the OS, application version and dependencies. Dependencies are managed either by including the dependency as part of the title or by including it as a separate title. If the dependency is a separate title, the relationship is defined in the ED file and is incorporated into the Capsules via environment variables. For example, an ED file could define a particular application version that requires a specific version of Python, in which case the ED file would set the SBGrid Python version variable, which would in turn be used to set the PYTHONPATH and LD_LIBRARY_PATH. These variable settings would only be accessible to the application environment. Additionally, dependency information in the ED files feeds the SBGrid installation process, thereby guaranteeing that the required dependency titles are installed.

Testing by the SBGrid curators is never complete given the magnitude of options to execute applications and interconnect them into unique workflows. The logic within each of the ED files has been developed over many years, and gradually refined with feedback from the SBGrid community. After the SBGrid collection has been distributed to SBGrid users, application errors may still occur, but they are usually quickly reported/flagged and resolved by SBGrid curators, often in coordination with software developers, and the updated versions are distributed to users. Statistics from the first 45 days of 2024 reveal that the root cause of user-submitted tickets predominantly involved dependency and version compatibility (26.2%), followed by installation issues (15.4%) and usage knowledge gaps (8%). The ED files persist throughout the life of the applications, thereby ensuring that the corresponding corrections, if applicable, are applied to previous and future versions of the particular title. With approximately 500 applications in each branch of the SBGrid collection, SBGrid maintains one ED file per title or approximately 1000 ED files between Linux and Mac OSs. Additionally, these ED files are not specific to particular versions of software titles but rather contain extensive logic that supports all versions of each software title provided through SBGrid.

With the ED files in place and before the SBGrid collection is synchronized with all SBGrid member laboratories globally, the SBGrid deployment scripts generate CR scripts and copy them to a single distribution directory. The CR scripts are generated for all executables in the SBGrid collection, but they are derived from the title-specific ED files and are virtually identical for all executables that map to a particular title. Importantly, the CR scripts are not specific to a particular version of the software titles. They contain the full version logic included in the corresponding ED file and ultimately execute version-specific executables when called by the end user. Notably, the CR scripts provide immediate, zero-configuration access to all software titles in the SBGrid collection, allowing users to select and immediately execute titles and versions of software as they navigate through the complex landscape of structure determination.

**Figure 5:**
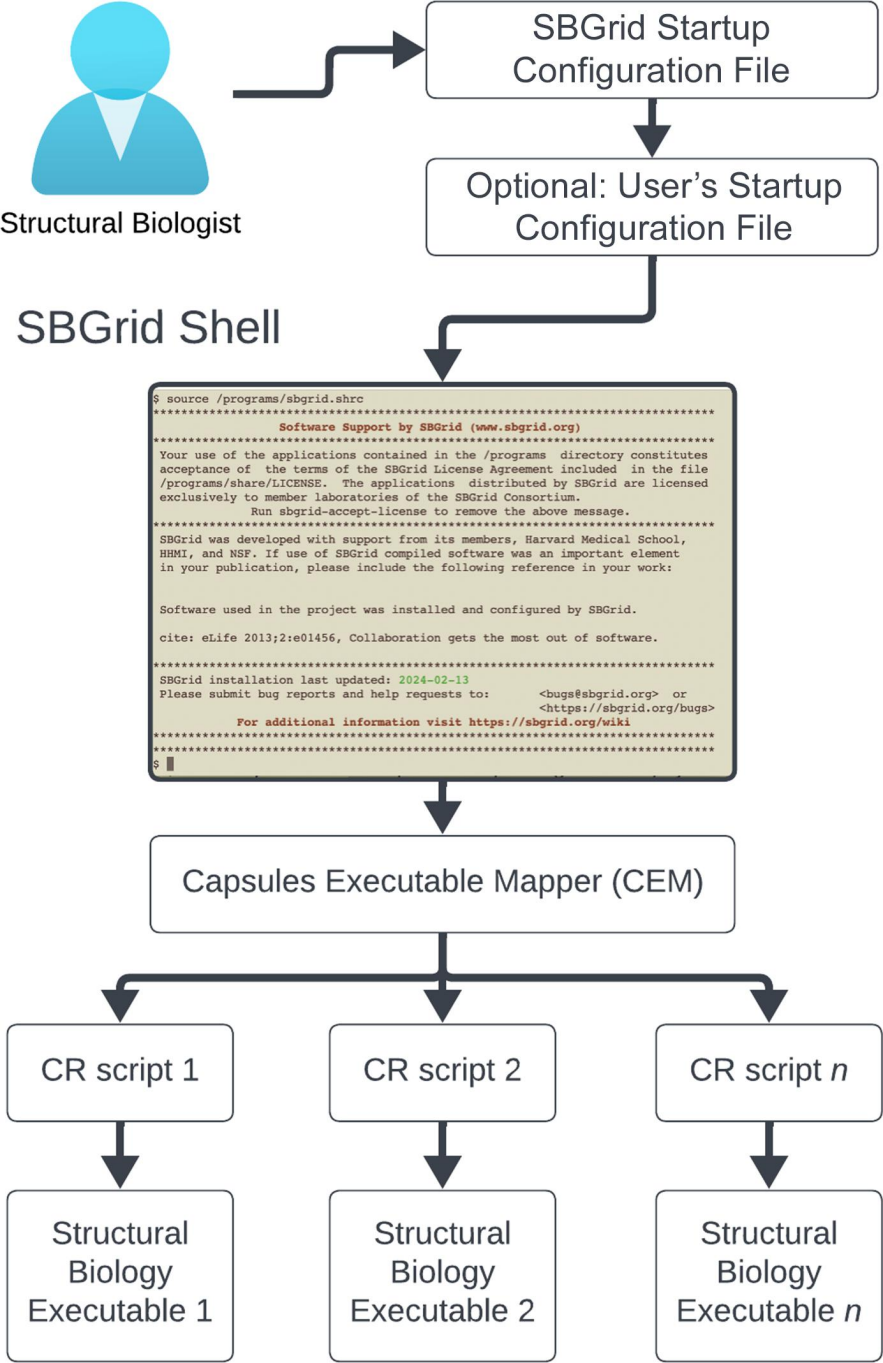
Comprehensive illustration of the Capsules initiation and application execution process on computers in SBGrid Consortium laboratories (assuming that SBGrid has been already pre-installed with the SBGrid installation manager). This figure showcases the streamlined, single-step initiation method where users begin by executing an OS-agnostic SBGrid Startup Configuration file, compatible with bash/zsh and tcsh for various UNIX shell environments. Users can then incorporate a user-specific configuration file (.sbgrid.conf) to fine-tune software versions and preferences. Once the environment has been activated, users gain immediate, zero-configuration access to an extensive collection of executables, facilitated by the SBGrid Capsules Executable Mapper (CEM), which efficiently resolves naming conflicts between executables with identical names, ensuring smooth execution of the desired executable. This process underscores the ease-of-use approach of the SBGrid system to managing distributed computing resources.

The initiation of Capsules on systems that have the SBGrid collection installed is a single-step process (Fig. 5 ▸). A user simply executes the source command on an OS-agnostic SBGrid startup configuration file (/programs/sbgrid.[shrc|cshrc]). Bourne and C-shell versions of this file are available to support widely used UNIX shell environments. The simplicity of accessing the SBGrid collection is important, giving SBGrid users the ability to load and unload the SBGrid environment on demand. This is particularly critical for new users to support the transition time between their earlier configuration and SBGrid. Initiating SBGrid environments supersedes previous configurations; for example, variables such as PATH and LD_LIBRARY_PATH are set to SBGrid-specific values. However, the environment can always be reset by initiating a new shell session.

After sourcing the SBGrid startup configuration file, users can optionally include their custom user configuration file (.sbgrid.conf). Within this file, users can define version and title selection variables to further fine-tune the environment to their needs. The most widely used configuration option is to lock a specific version of a software title. This option is particularly useful for titles that are frequently updated because users often wish to use just one software version for the duration of the project, or may wish to resurrect a particular version to reproduce computations at a later date.

Zero-configuration access to all SBGrid programs is facilitated through branch-specific executable directories, which between the Linux and macOS branches contain 56 197 CR scripts. Storing all executable scripts in a single location bypasses the need to create an ever-expanding executable PATH variable, which is limited to a theoretical value of 32 760 characters and is not adequate to support our growing SBGrid collection. With the new Capsules environment, this constraint is eliminated. The number of CR scripts per directory is virtually unlimited (for example 2^64^ under the XFS journaling file system), and therefore users can easily access all executables in the ever-growing SBGrid collection.

CR scripts are called to run specific executables. CR script mapping is possible because most executable programs in the SBGrid collection have unique names and the executable program calls map to the most recent version of a given software title. Calling executable model_angelo, for example, maps to the CR script model_angelo, which executes the most recent version available for the user’s OS and initializes the environment.

Given the size of the entire SBGrid collection, namespace overlaps between individual executables do occur. Different executables could originate from variations of the same version of a given application that is packaged and optimized by different developers, such as with *Coot* (Emsley *et al.*, 2010 ▸), which is available as a standalone title or bundled with other software titles, such as *CCP4* (Agirre *et al.*, 2023 ▸) or *CCP-EM* (Wood *et al.*, 2015 ▸; Burnley *et al.*, 2017 ▸). Executables with the same name might also be different applications that originate from different developers. For example, an executable *refine* is distributed with *BUSTER* (Bricogne *et al.*, 2023 ▸) by Global Phasing, where it is used to refine macromolecular coordinates against X-ray data, and a different *refine* is distributed with the cryoEM application *EMAN* (Ludtke, 2010 ▸), where it is used to refine coordinates against cryoEM data. Where two or more executables have the same name in the SBGrid collection, the overlap is resolved by the Capsules Executable Mapper (CEM; Fig. 5 ▸). CEM determines a list of candidate titles that contain the requested executable. From this candidate list, CEM eliminates titles based on the selection of the user, site, SBGrid’s default and ultimately directory order.

### 2.3. Capsules Configuration Language: CCL

The Capsules Software Execution Environment leverages an SBGrid-developed Capsules Configuration Language (CCL). This language consists of 41 directives that correspond to common shell programming paradigms and can be trans­piled into shell configurations that are compatible with either Bourne or C-style shells (Supplementary Table S2). The original monolithic execution environment utilized RC files, a widely adopted convention in Linux and Unix-like operating systems referred to as ‘Run Commands’ (Pouzin, 1965 ▸), to generate static shell configurations for shells such as tcsh and bash. With the move to Capsules, the CCL transpiler was extended to support the slightly different bash syntax used for runtime evaluation.

The Capsules framework uses the CCL and ED files during both installation and execution. CR scripts are generated based on requirements defined by CCL directives in the ED files. Capsules also read the CCL configurations from ED files during execution, which allows them to dynamically configure and customize the software environment on a per-executable basis. This customization can include version logic, setting or unsetting environment variables, adding custom command-line arguments or even invoking other executables.

### 2.4. Transition to Capsules

The gradual transition from the original monolithic environment to Capsules began in April 2016. By August 2019, Capsules were the default operational mode across the entire consortium. The development of the Capsules paradigm was structured to maintain backward compatibility with the monolithic paradigm. It was important to deploy Capsules with minimal interruption and with ongoing feedback from the structural biology community. In the early phases of the migration, access to Capsules was offered on an opt-in basis. Users could elect to switch to Capsules by simply setting an environment variable before sourcing the sbgrid.shrc or sbgrid.cshrc file. Later, this process was replaced by a small utility application, *sbcap*, which was utilized to turn the new environment on and off. In operational contexts managed by SBGrid personnel, particularly in training scenarios, Capsules were used to elicit tangible feedback from real-world applications. This strategy, bolstered by a wealth of empirical data, has conclusively established the Capsules method as the consortium’s definitive operational paradigm, effectively replacing the traditional deployment methods for all users.

### 2.5. Capsules support reporting functionality

**Figure 6:**
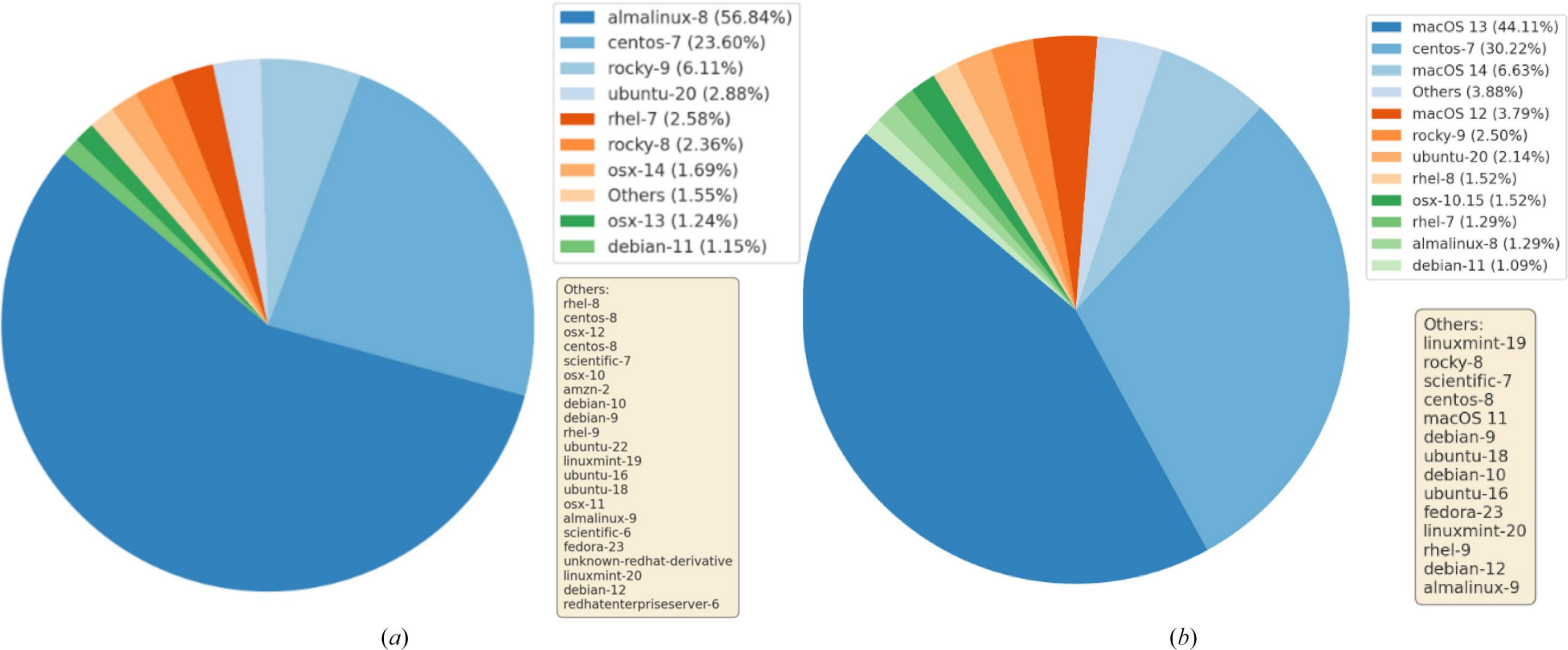
Capsules logging of local and global application usage. The composite figure of two pie charts illustrates the distribution of host operating systems running SBGrid jobs in January 2024 on on-premises and cloud-based computers supporting the SBGrid Consortium laboratories. It shows operating systems with shares over 1% and an aggregate ‘Others’ category that accounts for operating systems each under 1%. (*a*) The pie chart illustrates the percentage of user-hosting operating systems, filtered by unique hosts, running approximately four million SBGrid jobs in January 2024. (*b*) The pie chart shows OS usage by jobs running a molecular-visualization application in January 2024. The log analytics reveal that more users run the molecular-visualization application on Mac than on Linux. Notably, CentOS-7 maintains the second-largest share in both (*a*) and (*b*) despite its declining usage following the announcement that it will reach end of life (EOL) in June 2024, reflecting its prior prominence in high-performance computing and scientific computing environments due to its long-term stability.

Capsules allow SBGrid to implement features beyond addressing user-encountered challenges with application-required environments. Perhaps most importantly, Capsules introduced support for software-application monitoring and reporting capabilities. Capsules capture various anonymized metrics for each SBGrid executable, which allow software curators to monitor the aggregate use of individual software titles and versions of executables as well as the runtime and exit status of executables. The curators can also visualize the aggregate distribution of computational environments that are utilized by SBGrid users, including the breakdown between Linux and macOS, as well as more specific information about versions of the supported OSs (Fig. 6 ▸ and 7 ▸).

Access to the aggregate reports helps SBGrid to strategically prioritize software support. More effort is directed to optimizing and rolling out new versions or software applications that are more widely utilized by the consortium.

SBGrid’s general policy is to default to the most recent version of the application immediately after it is released to the structural biology community. While SBGrid users have the option to lock their configuration to earlier versions of updated applications, Capsules reports reveal that most users routinely advance to the new versions of software. This finding demonstrates one of the major benefits of SBGrid, which is to provide the structural biology community at large with almost immediate access to rapidly advancing software technologies.

The Capsules reporting system also has the potential to provide individual users, local system administrators and software developers with access to usage data. Through the local configuration options, users and local system administrators are able to capture usage within local users’ directories, providing an electronic diary of project progressions. Software developers might also request reports about the use of their software applications to support their development efforts and applications for funding support.

### 2.6. SBGrid installation manager and modular distribution

The entire SBGrid collection, together with the corresponding ED (Environment Definition) files, CR (Capsules Runtime) scripts and the CEM (Capsules Execution Mapper), continues to be distributed to the SBGrid consortium laboratories through the SBGrid *sync* system (Morin *et al.*, 2013 ▸). *Sync* effectively pushes the SBGrid collection to file servers at member institutions, which typically then provide access over the Network File System (NFS) to Linux and Mac computers in SBGrid-affiliated laboratories. Between all of the monthly upgrades and additional unscheduled patching to all 104 sites, the *rsync* process pushes out on average 30 TB per month across all of the SBGrid sites, or 290 GB per site. This includes all SBGrid pre-compiled software and supported libraries, but would typically exclude tarballs, compressed files and any other code that is not supportive of immediate execution. The installation system typically runs on a regularly scheduled basis and can upgrade local installations whenever new changes on the SBGrid server are put into place. The entire process is completely transparent to the end users. No additional steps are required to run the software.

**Figure 8:**
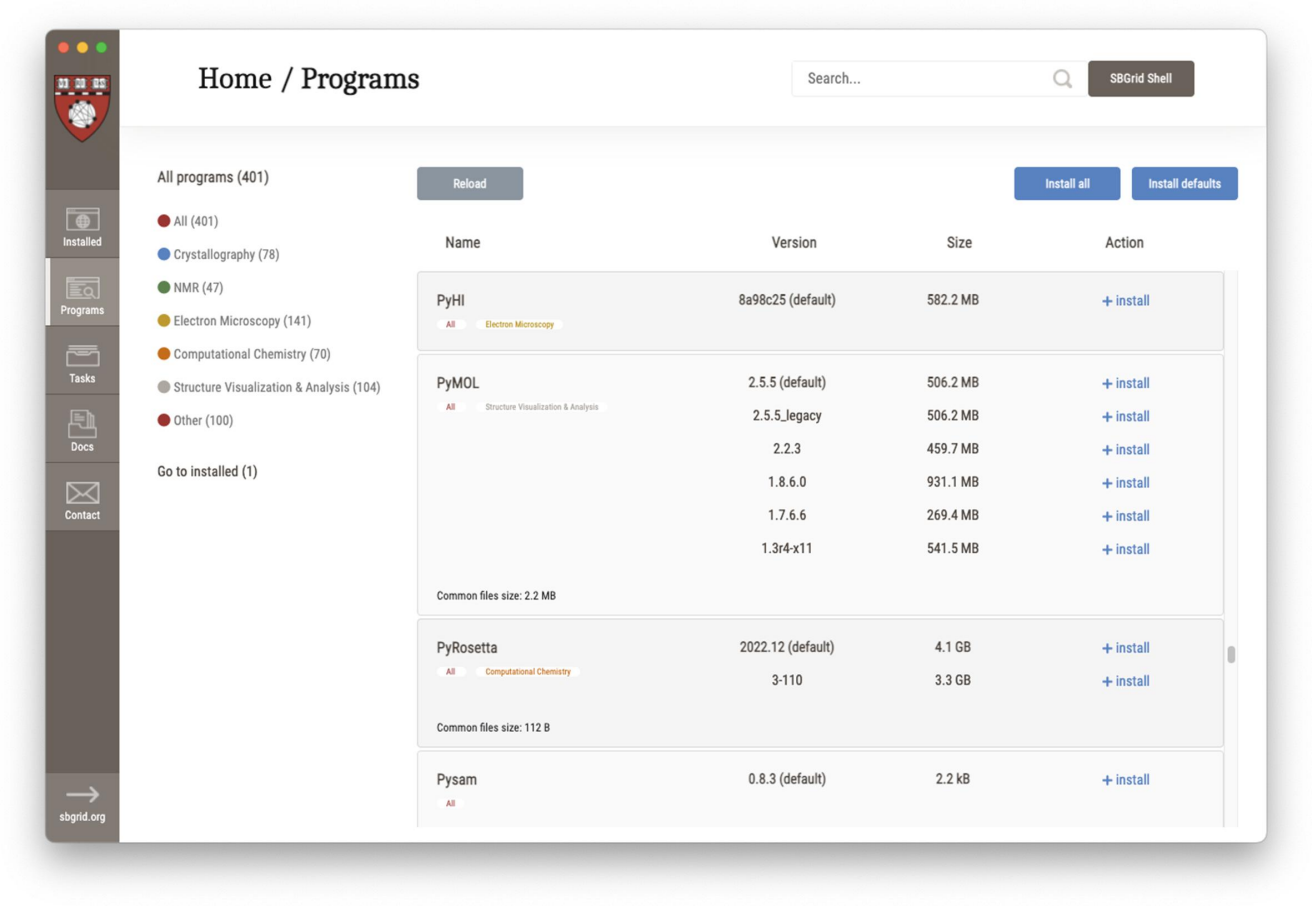
The SBGrid installation manager provides an intuitive and user-friendly interface that serves as the primary platform for both individual users and system administrators to manage SBGrid software. An efficient, secure and scalable tool for installation and maintenance tasks, it supports a variety of functions, including the activation of new installations with provided credentials, diagnostics and installing and updating, as well as removing, software.

Over the past few years, a new more personalized approach to software installations has been introduced in the form of our SBGrid installation manager. This graphical user interface application (Fig. 8 ▸) resides on members’ computers and allows users to pick and choose individual software titles for installation. The SBGrid installation manager has a similar look and feel to the software listing on the SBGrid website. Users can browse the SBGrid collection, select individual versions for installation and perform updates of previously installed software titles. It relies on personal credentials that are associated with the user’s laboratory, thus ensuring access to a selection of software titles appropriate to their institution type. Site administrators and advanced users can also benefit from the extensive command-line interface, which incorporates and expands on functionalities offered through the *sync* method. Moreover, support for software installation over HTTPS provides encryption in transit and enables sites that block non-HTTP ports by default.

Use of the SBGrid installation manager has rapidly expanded over the last few years, with 3752 installations currently supported. This new modular approach to software synchronization streamlines the task of software management to support the diverse range of license configurations and tailored installation types required to meet the needs of SBGrid laboratories. The modularity ensures that the SBGrid deployment is capable of accommodating a repository of thousands of software titles and versions while also offering the flexibility to install small subsets of applications. It is therefore particularly suited to meet the requirements of individual users.

### 2.7. MultiGrid: scale-up to other domains

The software titles in the SBGrid collection are typically limited to applications that directly or indirectly support the unique needs of structural biology experimentalists. However, laboratories that primarily utilize NMR, X-ray crystallography and cryoEM in their research routinely venture to other fields of computational biology. In those cases, they typically prefer to reuse the existing CPU and GPU resources for computing and the familiar SBGrid platform for software access.

To support this emerging need, we recently developed the BioGrids collection, a separate collection of scientific software more broadly focused on computational biology that has rapidly expanded to over 700 titles. Providing support to parallel scientific domains in a single software tree necessitated the extension of Capsules and the development of multigrid functionality. Using the grid membership CCL directive setGrid, a single software title can be assigned to any number of defined grids. The tools that operate on the ED files were also extended to create parallel environments for all of the defined grids. While there are only two grids supported at this time, the enhancement was designed to allow for any number of grids. The decision to extend rather than duplicate SBGrid is advantageous because a software package relevant to multiple grids is only installed once in the general build-and-test environment and only one ED file is needed. Adding or removing an application from a grid is performed with a simple edit of the application.rc file. Using this system, applications that are relevant to multiple domains are curated once, and for sites with multiple collections installed one software tree supplies both grids. End users can select a grid easily by accessing the appropriate startup environment, and switching grids is a simple environment change.

## 3. Discussion

In this manuscript, we have presented the new Capsules system developed by our team, tailored specifically for structural biology. This system enhances the management and distribution of software within the SBGrid collection. It promotes access to essential computational tools across a diverse range of computational resources and contributes meaningfully to the dissemination of knowledge and tools, improving the accessibility and usability of computational resources in research. The adoption of Capsules over the previous SBGrid monolithic shell environment model marks an advancement in software management, addressing challenges with the scalability and complexity of large collections of scientific software. The Capsules technology enables SBGrid to support installations that range from a single application to hundreds of applications with multiple versions in a distributed, conflict-free environment. By isolating the runtime environments of individual applications, our method simplifies application use and effectively addresses the environment- and version-specific conflicts that were prevalent in the monolithic model.

As it is for other disciplines, software is essential for modern structural biology research. The FAIR principles (Findable, Accessible, Interoperable and Reusable; Wilkinson *et al.*, 2016 ▸), and their current adaptation into FAIR4RS (Barker *et al.*, 2022 ▸), can serve as an impetus to treat scientific software as scholarly objects in their own right and to advance research effectiveness. The Capsules environment has a foundational role in the SBGrid technology stack in implementing these principles. The ‘Findable’ principle is addressed through the SBGrid website that serves pages to human visitors. When community standards for machine-readable metadata become available, this information can be added to software ‘landing pages’, similar to the way that data-set pages were added to the SBGrid Data Bank. The ‘Accessible’ principle is addressed by the SBGrid installation manager, which was developed specifically to make software in the SBGrid collection accessible to consortium members broadly. Although archived software versions are no longer provided by SBGrid and are not immediately accessible, SBGrid software curators can reinstall them upon request. The Capsules environment supports the ‘Interoperable’ principle by allowing the incorporation of different software components into scientific workflows or pipelines and by isolating and controlling dependencies; however, support for community-standard file formats and APIs (application programming interfaces) remains the domain of application developers. The principle of ‘Reusability’ is illustrated in the Capsules environment and its capacity to allow software installation and usage on systems other than those on which it was developed, with minimal effort from researchers.

Security of software is increasingly critical in the face of sophisticated cybersecurity threats that have impacts on educational and research institutions. Ransomware attacks on institutions like that on the UCSF School of Medicine, which resulted in a substantial ransom payment to regain access to encrypted files (Tidy, 2020 ▸), underscore the direct threats to academic research integrity and data security. The increasing prevalence of ransomware-as-a-service further complicates the cybersecurity landscape, making robust software security measures an essential component of any education and research computational infrastructure (Government Accountability Office, 2021 ▸; Koomson, 2021 ▸). Two basic factors of the current Capsules implementation are relevant to security: (i) all titles and dependencies are installed outside the context of the base OS, so vulnerability present in the SBGrid collection would not impact system services (for example, if the recent XZ CVE-2024-3094 had been present in the SBGrid collection, it would not have resulted in a vulnerable SSHD service), and (ii) all titles are tested, and known to work, when run under an account without elevated system permissions or privileges. Although Capsules were not initially designed to address security concerns, the self-contained execution environment designed for reliable and reproducible research computing could be used as an entry point to enhance the security of scientific computations. OS-level application-sandboxing technologies allow sub-account-level execution restrictions (for example, allowing read/write access to a processing directory but blocking read access to other areas of a user’s home directory and arbitrary network access). Tools for sandboxing can be complex to configure, and require detailed knowledge of how a program behaves during normal operation; future development of the Capsules environment could allow end users to run in the context of a sandbox determined during software curation. Although it would require additional infrastructure and development, the Capsules environment could also perform out-of-band verification of pre-calculated code signatures, blocking the execution of modified software.

The ability of Capsules to capture anonymized and aggregate information about software use can support efforts aiming to improve the research-software ecosystem. SBGrid already uses aggregate software-utilization information to prioritize software updates, elevate popular titles and effectively manage the retirement of unused applications or outdated versions. These detailed insights into application usage patterns also inform the SBGrid team and facilitate the development of customized educational content such as webinars, workshops and seminars that evolve with the changing needs and preferences of the scientific community. These global software usage metrics are also available to contributing software developers upon request and could support future refinement of tools to address real-world usage. Looking ahead, the possibility of capturing the provenance of software execution for completed projects, in alignment with features implemented in software suites such as *CCP4* (Agirre *et al.*, 2023 ▸) and *Phenix* (Liebschner *et al.*, 2019 ▸), addresses the ongoing crisis in reproducible research and positions Capsules as a viable solution in enhancing scientific rigor, offering one challenging but potentially impactful development opportunity.

Taken together, Capsules are now an integral part of the SBGrid platform, supporting streamlined access to the research-software ecosystem within the SBGrid collection in a range of computational environments. Initially developed for the discipline of structural biology, the development of the BioGrids collection demonstrates the generalizability of this approach. The versatility of Capsules allows their adoption across different scientific domains, potentially transforming software management and accelerating scientific research on a much larger scale. This expansion could influence how biomedical research is conducted, offering tailored solutions that meet the specific needs of various disciplines.

## Supplementary Material

Supplementary Figure S1, Table S2 and caption to Table S1. DOI: 10.1107/S2059798324004881/gm5107sup1.pdf

Supplementary Table S2. DOI: 10.1107/S2059798324004881/gm5107sup2.csv

## Figures and Tables

**Figure 7:**
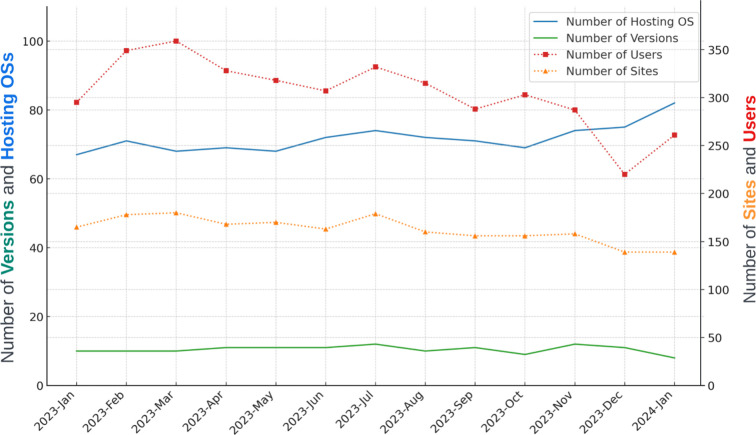
Monthly usage metrics for the same molecular-visualization title as reported in Fig. 6 ▸(*b*). This figure charts the usage of a molecular-visualization application from January 2023 to January 2024. It shows the number of application versions used, including minor releases, and the number of unique host OSs on which the application runs. These data points are plotted on the left *y* axis as solid lines. On the right *y* axis, the figure displays the monthly count of sites and users engaging with the application, indicated by dashed lines.
