# Supplementary material for: Introduction of the Capsules environment to support further growth of the SBGrid structural biology software collection
Source: Acta Crystallogr D Struct Biol. 2024 Jun 4;80(Pt 6):439–50. doi: 10.1107/S2059798324004881 (PMC11154594; doi:10.1107/S2059798324004881)
Supplement: Supplementary file 1 [file d-80-00439-sup1.pdf]

STRUCTURAL
BIOLOGY

Volume 80 (2024)

Supporting information for article:

Introduction of the Capsules environment to support further growth of the SBGrid structural biology software collection

Carol Herre, Alex Ho, Ben Eisenbraun, James Vincent, Thomas Nicholson, Giorgos Boutsioukis, Peter Meyer, Michelle Ottaviano, Kurt L. Krause, Jason Key and Piotr Sliz

```
# C Herre dbb7c5e 2023-10-28 23:23:18 -0400
# Copyright © 2023 SBGrid Consortium.  All rights reserved.
#
setGrid "sbgrid"
setVersion PHENIX 1.20.1-4487 "1.21rc1-5107 1.21rc1-5049 1.21rc1-5015
dev-4788 1.20-4459 1.19.2-4158 1.19.1-4122 1.18.2-3874"
setEnv PHENIX_DEP_SET "rosetta ccp4 neggia"

setDep 1.21rc1-5049 "pymol@2.2.3"
setDep 1.21rc1-5015 "pymol@2.2.3"
setDep dev-4788 "pymol@2.2.3"
setDep 1.20.1-4487 "pymol@2.2.3"
setDep 1.20-4459 "pymol@2.2.3"
setDep 1.19.2-4158 "pymol@2.2.3"
setDep 1.19.1-4122 "pymol@2.2.3"
setDep 1.18.2-3874 "pymol@2.2.3"

ifSet __SBCAP
# SCHRODINGER Breaks refinement
  ifSet SCHRODINGER
    unsetEnv SCHRODINGER
  endTest

  setEnv SB_PATH "${verdir}/phenix-${appvar}/build/bin:"
  setEnv SBCAP_PATH "${verdir}/phenix-${appvar}/conda_base/bin"
  setEnv PHENIX "${verdir}"
  setEnv LIBTBX_BUILD ""
  setEnv PHENIX_VERSION "${appvar}"
  setEnv LIBTBX_OPATH ""
  setEnv LIBTBX_TMPVAL ""
  setEnv PHENIX_SUPPRESS_VERSION_CHECK true

  fileTest '-e' '/programs/x86_64-linux/phenix/phenix.prefs'
    setEnv PHENIX_GUI_DEFAULT_PREFS /programs/x86_64-
linux/phenix/phenix.prefs
  endTest

elseTest
  setEnv PATHS_FIRST ${verdir}/bin.capsules
endTest
```

(a)

```
#!/bin/bash

# Copyright © 2020 SBGrid Consortium.  All rights reserved.
#
# This is a capsule generated for phenix.setup_tutorial.
# For help on capsules, run: phenix.setup_tutorial --sbapp:H
# For path to phenix.setup_tutorial, run: phenix.setup_tutorial --sbwhich
#
# To learn more about capsules visit:  https://sbgrid.org/wiki/capsules

SB_APPNAME=phenix
SB_EXECNAME=$(basename $0 | sed s%__"$SB_APPNAME"$%%)
[[ -f $0 ]] && SB_EXECDIR=$(dirname $0)
[[ -L $0 ]] && SB_EXECDIR="$(cd $(dirname $0) && pwd)/$(dirname $(readlink
$0))"

. "$SB_EXECDIR"/capsule_header.sh

# set GRID to none if not selected by user startup
export GRID=${GRID:-"none"}

SB_CAPSULE_VERSION=x86_64-linux-01.05.23_15805_70857b2
SB_CAPSULE_CREATED=x86_64-linux-01.05.23_15805_70857b2

export PATH="$SBCAP_GRIDBIN":/usr/bin:/bin:/usr/sbin:/sbin:/usr/local/bin

. "$SB_CAP/lib/includes.sh"

read_rc
read_dep_rc

sb_getopts "$@"

start_timer
run_job "$@"
stop_timer

gather_metrics
report_metrics

exit "$SB_JOBEXIT"

# grid member: "sbgrid"
# part of: phenix 1.20.1-4487
```

(b)

Figure S1 (a) An example ED file for the *Phenix* title. This ED file contains title-specific environment requirements and is used in the SBGrid curation process to generate the CR scripts. (b) An example of an SBGrid CR script for the Phenix (Liebschner *et al*., 2019) application is provided. The CR script, named `phenix__phenix`, is specifically crafted for the `phenix` executable from *Phenix*, the title. The CR

script is automatically generated as part of the SBGrid installation process. The CR script uses the RC custom directives to manage the environment setup and operational requirements of the executable.

Table S1 Comprehensive Table of all SBGrid applications, with the following information (CSV format Excel spreadsheet)

- Software Title
- Country of Origin
- Production Date
- Linux/Mac Version data
- License Type
- Was title used in the first 8th months of 2023: yes/no
- For 50 titles that were randomly selected from above, a distribution format for the title
- For the 10 most frequently used titles, indicate the programming language used to develop the application

Table S2 Overview of Capsules Configuration Language (CCL) 41 custom directives with corresponding use cases and directive descriptions. A) The use case is for setting up Capsules. B) The use case is for the process of generating the CR script. C) The use case is for creating shell RC files that contain commands and settings for initializing and configuring software and user environments.

Directive	A	B	C	Description
addEnv	x	x	x	Adds argument to the defined environment variable in a colon-separated list.
addSub	x		x	Supports placing the title directory in a subdirectory of the platform directory. Providing a mechanism for partitioning the SBGrid collection based on specific domain criteria. Used today to isolate operational-specific titles like curl and rsync.
beprint			x	Prints the provided argument in a format that can be evaluated in a bash shell.
bprint	x	x	x	Prints the provided argument to be used in setting up bash shell initialization.
comment			x	Prints a comment in the generated shrc and cshrc files. General comments in an rc file are not included in the generated files.
cprint			x	Prints the provided argument to be used in setting up csh/tcsh shell initialization.
customVersion	x		x	Same as setVersion but allows for custom naming of the version variable.
echoErr	x		x	Prints a message provided as an argument to standard error.
echoOut	x		x	Prints a message provided as an argument to standard output.
elseIf	x	x	x	Alternate processing when the initial if is not met and adds a new if condition.
elseTest	x	x	x	Alternate processing when the if condition is not met.

endTest	x	x	x	Terminates any of the conditional statements such as if64bit, osGt, elseTest, elseIf, ifSet, fileTestetc.
fileRead	x		x	Sources the files provided as arguments and applies the contents of the file to the environment. Requires two files one for bash shell and one for csh.
fileTest	x		x	Provides a conditional operation to check for the argument provided test condition of the argument provided filename. Commands following this directive are processed if the test condition operation is matched. Requires endTest, elseTest or elseIf for marking the end of the process block.
if64bit	x		x	Provides a conditional operation based on system architecture that is 64-bit. Commands following this directive are processed if the architecture is 64-bit. Requires endTest, elseTest or elseIf for marking the end of the process block.
ifarm64	x		x	Provides a conditional operation based on system architecture is arm64. Commands following this directive are processed if the architecture is arm64. Requires endTest, elseTest or elseIf for marking the end of the process block.
ifnot64bit	x		x	Provides a conditional operation based on system architecture that is not 64-bit. Commands following this directive are processed if the architecture is not 64-bit. Requires endTest, elseTest or elseIf for marking the end of the process block.
ifnotarm64	x		x	Provides a conditional operation based on system architecture is not arm64. Commands following this directive are processed if the architecture is not arm64. Requires endTest, elseTest or elseIf for marking the end of the process block.
ifSet	x		x	Provides a conditional operation based on if the argument provided variable is set to any value including the empty string. Commands following this directive are processed if the variable is defined. Requires endTest, elseTest or elseIf for marking the end of the process block.
ifUnset	x		x	Provides a conditional operation based on the argument provided variable is not set or initialized. Commands following this directive are processed if the variable is not defined. Requires endTest, elseTest or elseIf for marking the end of the process block.
ifVersion	x	x	x	Provides a conditional operation based on the argument provided version matching the defined title version variable. Commands following this directive are processed if the title version variable matches the argument. Requires endTest, elseTest or elseIf for marking the end of the process block.
indentPrint			x	Used to format the output of the generated shrc and cshrc files to increase the readability of the generated files.
iprint			x	Generates an installer definition file that can be used by the sbgrid_installer to create the install on another host.
modprint			x	Creates Lmod Environment Module System compatible lua files for all the installed titles.
osEq	x		x	Provides an equality comparison of the provided argument to the OS version running on the system. Commands following this directive are processed if the OS versions are equal. Requires endTest, elseTest or elseIf for marking the end of the process block.
osGt	x		x	Provides a greater-than comparison of the OS version running on the system and the provided argument. Commands following this directive are processed if the OS version is greater-than the argument. Requires endTest, elseTest or elseIf for marking the end of the process block.

osLt	x		x	Provides a less-than comparison of the OS version running on the system and the provided argument. Commands following this directive are processed if the OS version is less-than the argument. Requires endTest, elseTest or elseIf for marking the end of the process block.
runDeploy		x		Runs an executable provided as an argument during the SBGrid deployment pipeline process.
runIt	x		x	Runs the executable that is provided as an argument.
setAlias		x	x	In category 1, it creates a shell alias in the user’s general environment.<br>In category 2, it creates a script that matches the alias definition.
setDep	x		x	Defines title and version specific dependencies between titles resulting in the dependency titles’ variables being automatically set to the required versions. This allows for a title with very version specific dependencies to ensure that the executable will always access the correct version.
setEnv	x	x	x	Defines an argument-provided environment variable to be set to an argument provided value.
setGrid		x	x	Defines the set of grids that should contain the title. Grids are provided in a single list argument.
setGrids		x	x	Alias of setGrid.
setName	x		x	Defines appname when it cannot be determined programmatically.
setVersion	x	x	x	Defines the version variable, default version and other available versions. The version variable is used to select the title’s version that should be used.
testDev	x			Automated testing tools for curation specific testing. These tests can be used by curators to validate an installation.
testSane	x			Automated testing tool for sanity tests. The testSane tests are run during the deployment pipeline.
unsetAlias		x	x	Removes an alias definition from the environment.
unsetEnv	x	x	x	Removes an environment variable.
versionRC	x	x		Provides a mechanism for splitting the ED file into version specific files contained in the version directory. This helps reduce the complexity of the ED files by separating the version-specific details. The benefit is realized when a version directory is removed or recovered since all of the version environment details are removed/recovered with the directory removal/recovery.
